# Acetylcholine versus cold pressor testing for evaluation of coronary endothelial function

**DOI:** 10.1371/journal.pone.0172538

**Published:** 2017-02-16

**Authors:** Ahmed AlBadri, Janet Wei, Puja K. Mehta, Sofy Landes, John W. Petersen, R. David Anderson, Bruce Samuels, Babak Azarbal, Eileen M. Handberg, Quanlin Li, Margo Minissian, Chrisandra Shufelt, Carl J. Pepine, C. Noel Bairey Merz

**Affiliations:** 1 Cedars-Sinai Heart Institute, Los Angeles, California, United States of America; 2 Division of Cardiovascular Medicine, University of Florida College of Medicine, Gainesville, Florida, United States of America; 3 Biostatistics and Bioinformatics Research Center, Cedars-Sinai Medical Center, Los Angeles, California, United States of America; University of Bologna, ITALY

## Abstract

**Background:**

Assessment of coronary endothelial function with intracoronary acetylcholine (IC-Ach) provides diagnostic and prognostic data in patients with suspected coronary microvascular dysfunction (CMD), but is often not feasible due in part to the time and expertise needed for pharmacologic mixing. Cold pressor testing (CPT) is a simple and safe stimulus useful for either invasive or non-invasive endothelial function testing and myocardial perfusion imaging but has not been specifically evaluated among symptomatic women with signs of ischemic heart disease (IHD) who have no obstructive coronary artery disease (CAD).

**Methods:**

163 women with signs and symptoms of IHD and no obstructive CAD from the NHLBI- Women’s Ischemia Syndrome Evaluation-Coronary Vascular Dysfunction (WISE-CVD) study underwent coronary reactivity testing with a Doppler flow wire (FloWire^®^ Volcano, San Diego, CA) in the proximal left anterior descending artery. Coronary artery diameter and coronary blood flow (CBF) assessed by core lab using QCA before and after IC-Ach (18.2 μg/ml infused over 3 minutes) and during CPT.

**Results:**

Mean age was 55 ± 12 years. Rate pressure product (RPP) in response to IC-Ach did not change (baseline to peak, P = 0.26), but increased during CPT (363±1457; P = 0.0028). CBF in response to CPT was poorly correlated to IC-Ach CBF. Change in coronary artery diameter after IC-Ach correlated with change after CPT (*r* = 0.59, P<0.001). The correlation coefficient was stronger in subjects with coronary dilation to IC-Ach (*r* = 0.628, P<0.001) versus those without dilation (*r* = 0.353, P = 0.002), suggesting that other factors may be important to this relationship when endothelium is abnormal.

**Conclusions:**

In women with no obstructive CAD and suspected CMD, coronary diameter changes with IC-Ach and CPT are moderately-well correlated suggesting that CPT testing may be of some use, particularly among patients with normal endothelial function, however, not an alternative to IC-Ach for diagnosis of coronary endothelial dysfunction.

## Background

Women with signs and symptoms of myocardial ischemia are diagnostic and therapeutic challenges. This is due, in part, to a low prevalence of obstructive coronary artery disease (CAD), resulting in relatively high costs of care related to repeated testing, repeated hospitalizations, and disability [1]. Although knowledge of the mechanisms explaining these findings is limited, impaired coronary reactivity (endothelial- and non-endothelial dependent functions) and coronary artery spasm have been proposed to contribute [2–9]. The endothelial-dependent component has been linked to atherosclerosis risk factors and pro-inflammatory processes [2, 4] as well as adverse clinical outcomes [4, 6]. Consequently, various interventions, primarily pharmacologic, have been used to evaluate coronary artery endothelial function and provoke coronary artery spasm during coronary reactivity testing (CRT) [9, 10].

Endothelial integrity is essential to normal vascular physiology, and its dysfunction is believed to be a critical factor in the pathogenesis of vascular disease and related adverse outcomes. When endothelium is functioning normally, acetylcholine (Ach) causes vasodilation by release of biologically active nitric oxide (NO) in sufficient quantity to counter the effect of Ach to directly activate muscarinic receptors on vascular smooth muscle cells. Whereas when endothelium is damaged, Ach causes vasoconstriction via direct stimulation of vascular smooth muscle muscarinic receptors. [11] The cold pressor test (CPT), a sympathetic reflexogenic stimulus, increases coronary resistance thereby reducing blood flow in patients with CAD, presumably by sympathetically mediated vasoconstriction. [12] CPT also increases myocardial oxygen demands, which results in metabolically mediated increases in coronary blood flow. This increase in blood flow results in an increase in shear stress at the endothelial surface to release NO from normal endothelium that contributes to coronary dilatation.

Intra-coronary Ach (IC-Ach) provides diagnostic and prognostic data in patients with suspected coronary microvascular dysfunction (CMD), but is often not feasible for due to the time and expertise needed for pharmacologic mixing. Furthermore, Ach cannot be given intravenously due to safety concerns so it cannot be used for noninvasive studies of endothelial function and CPT may offer this possibility if its relationship to endothelial function was better understood. Accordingly, we investigated the relationship between changes in coronary artery diameter in response to IC-Ach and CPT to determine the feasibility of using CPT for noninvasive provocative testing of endothelial function in future studies.

## Materials and methods

We studied 163 women enrolled in the National Heart, Lung, and Blood Institute (NHLBI)—Women’s Ischemia Syndrome Evaluation–Coronary Vascular Dysfunction (WISE-CVD) project who underwent consecutive IC-Ach and CPT. The WISE-CVD is a 2-center prospective, consecutive cohort project evaluating novel diagnostic techniques and pathophysiological mechanisms in women with suspected myocardial ischemia but without obstructive CAD. These women all had symptoms and signs of ischemic heart disease with clinically-indicated coronary angiography that revealed no obstructive CAD. Demographic data were recorded at baseline using standardized questionnaires. Cedars-Sinai Heart Institute and University of Florida sites participated in the coronary reactivity testing, and all coronary reactivity and angiographic data were read at core laboratories by expert readers (University of Florida, Gainesville, FL) masked to other participant data. The Institutional Review Boards at Cedars-Sinai Medical Center and University of Florida Medical Center approved the study, and all subjects gave written informed consent before study participation. IRB No: Pro00014906/Ame00016240.

### Assessment of coronary vascular function

Vasoactive medications were withdrawn for 48 hours before the procedure. Coronary reactivity testing was performed in an epicardial coronary artery free of obstructive CAD (<50% diameter stenosis). The left anterior descending (LAD) coronary artery was the preferred vessel, followed by the left circumflex coronary artery if anatomic issues prohibited safe access to the LAD. To assess blood flow velocity, a Doppler-tipped guidewire (0.014-inch FloWire, Volcano Corporation, California, USA) was advanced through the diagnostic catheter. Recordings were made once a stable Doppler signal in the proximal or mid vessel was obtained. Endothelial-dependent function was assessed with intracoronary (IC)—Ach, 0.182 μg/ml (10^-6^) at 2 mL/min for 3 minutes. This was followed by infusion of 18.2 μg/ml (10^-4^) over 3 min and then blood flow and pressure recordings were made and angiography was repeated to assess diameter [13, 14]. The 0.182 μg/ml IC-Ach was used for safety purposes and resulted in minimal changes in flow and diameter. Therefore, only the 18.2 μg/ml IC-Ach data are reported. Next CPT was performed by wrapping an ice pack around the forearm or placing an ice pack on the forehead for 2 minutes. Aortic pressure and heart rate were measured before and after applying the packs. After two minutes, angiography in a selected oblique projection was repeated. IC nitroglycerin was then infused and angiography repeated to assess non–endothelium-dependent epicardial coronary reactivity. [15]

### Quantitative coronary angiography and blood flow recordings

All angiography was done in a preselected oblique projection that minimized overlapping and foreshortening of the proximal to mid LAD. Angiograms and flow recordings were made at baseline and after administration of each vasoactive drug. A return to baseline flow velocity was documented before each new reactivity test. Pulsed-wave Doppler flow spectra were used to calculate time-averaged peak velocity (APV). Coronary cross-sectional area (CSA) was calculated from the diameter measured 5 mm distal to the tip of the Doppler wire. Coronary volumetric blood flow (CBF) was calculated with the equation CBF = CSAxAPVx0.5.[16] Epicardial responses to IC-Ach and CPT were assessed by measuring coronary diameter at baseline and after Ach infusion or CPT by quantitative coronary angiography. For quantitative coronary angiography, angiograms were analyzed by one investigator masked to all other patient data at the WISE-CVD angiographic core laboratory (University of Florida, Gainesville, FL) as described previously. [11]

### Statistical analysis

Summary statistics to describe the data are represented by the number and percentage, or mean and standard deviation where appropriate. Comparisons of heart rate, blood pressure and rate pressure product (RPP) at baseline and during the tests were performed using paired two sample t-test. Person’s correlation coefficient (r) was computed to estimate the strength of association between IC-Ach and CPT coronary artery diameter changes and CBF. For these analyses, the cohort was divided into groups based on angiographic findings: [Group 1] included those with normal response to IC-Ach infusion (i.e. dilation, normal endothelial response) and [Group 2] included those with an abnormal response to IC-Ach (i.e. no dilation or constriction, endothelial dysfunction). In addition, the sample was divided according to the CBF response (Normal: ≥50% increase in CBF from baseline in response to IC-ACH). All statistical analyses were performed in R version 3.2.3. Statistical significance was set a priori at p< 0.05.

## Results

Pertinent clinical characteristics are summarized in Table 1. The mean age was 55±12 years (range 26–82 years) with basal metabolic index (BMI) 30.4±8 kg/m^2^. The majority were postmenopausal, about half were obese (BMI ≥30 kg/m^2^); one third had a history of hypertension or family history of premature CAD. A history of diabetes was present in approximately one-tenth. During coronary reactivity testing, 82 (50.3%) subjects had a normal response to Ach defined by any dilation of the coronary artery after IC-Ach infusion and 73 (45%) had normal CBF defined as increase in CBF by at least 50% from baseline. Forearm CPT was performed in 94 subjects and forehead CPT in another 69 subjects. The RPP was taken as an index of cardiac work and as a measure of sympathetic stimulation with CPT. The increase in RPP was significant after CPT compared to baseline (363±1457) (p = 0.0028). The IC-Ach infusion had no significant effect on systemic hemodynamics at the concentrations given. None of the subjects developed any adverse events related to the tests or the procedure. CBF in response to CPT was poorly correlated to IC-Ach CBF (r = 0.23, P<0.01) while change in coronary artery diameter after IC-Ach moderately correlated with change after CPT (*r* = 0.59, P<0.001).

**Table 1:** Characteristics of women in the study (n = 163).

Age (years)	55±12
Smoking history, n (%) Current Previous smoker Never	4 (2%) 63 (43%) 79 (54%)
Family history of premature CAD, n (%)	52 (35%)
History of Diabetes, n (%)	16 (11%)
History of hyperlipidemia, n (%)	23 (16%)
History of hypertension, n (%)	50 (34%)
BMI (kg/m^2^)	30.4±8
LVEDP (mmHg)	15.23±5.9
CFR	2.77±0.71

CAD: coronary artery disease; BMI: body mass index; CFR = coronary flow reserve; LVEDP: left ventricular end-diastolic pressure; CFR: coronary flow reserve.

For age and body mass index, values are mean ± SD.

### Subjects with normal response to IC- Ach

**Fig 1:**
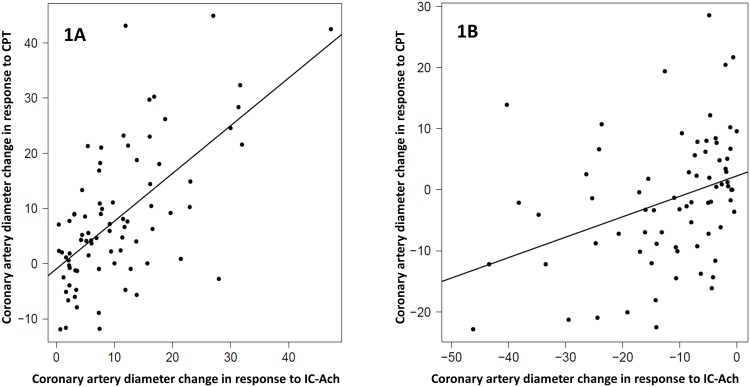
Correlations between in coronary artery diameter changes in response to acetylcholine (Ach) and cold pressor testing (CPT) (n = 163). Data are plotted as percent changes from baseline to post Ach or CPT. Subjects are stratified by their responses to Ach as either *normal* endothelial response (e.g. increase in diameter, 1A, left panel) or *abnormal* endothelial response (e.g. no change or decrease in diameter, 1B, right). Note that although correlations among both strata are statistically significant the correlation among those with normal endothelial function is strong (*r* = 0.628, P<0.001) but only moderate among those with abnormal endothelial function (r = 0.353, P = 0.002), supporting the suggestion that other factors contribute to this association.

Eighty-two women (50.3%) had normal response to IC-Ach infusion. During the CPT in these patients there was no significant increase in heart rate compared to baseline (P = 0.425), while there was a significant increase in aortic systolic pressure and RPP (P = 0.014 and P = 0.018, respectively). CBF in response to IC-Ach was poorly correlated to CPT CBF (*r* = 0.17, P = 0.16). At baseline, coronary blood flow velocity was 21.14±6.9 cm/sec and during CPT increased to 25.01±10.25 cm/sec (P = 0.007). Coronary artery diameter increased 10.45±9% in response to IC-Ach (Table 2). There was moderate correlation between Ach and CPT coronary artery diameter change (*r* = 0.628, P<0.001) (Fig 1A). Further vasodilation after CPT was obtained by IC nitroglycerin with change in coronary diameter of 20.2±12.95%.

**Table 2:** Systemic hemodynamics and coronary artery diameter changes in response to cold pressor test and IC-Ach (n = 163).

	Acetylcholine				Cold pressor test			
	Groups 1	P value	Group 2	P value	Groups 1	P value	Group 2	P value
**Change in heart rate (bpm)**	-0.49±6.77	0.54	0.82±6.41	0.27	0.64±6.92	0.43	0.84±9.33	0.45
**Change in SBP (mmHg)**	-2.05±13.61	0.20	-1.82±12.01	0.20	3.54±12.25	0.014	3.44±14.94	0.053
**Change in RPP (HRxSBP)**	-242±1208	0.09	-30.14±1194	0.83	339±1220	0.018	373±1693	0.064
**Change in coronary artery diameter (mm)**	10.45±9.00	<0.001	-12.25±11.73	<0.001	8.2±12.5	<0.001	-1.65±10.62	0.19

P values indicate change from baseline.

Group 1: participants who had normal response to IC-Ach defined as any dilatation of the coronary artery after the infusion compared to baseline diameter (i.e. normal >0% change in coronary artery diameter compared to baseline).

Groups 2: participants who had abnormal response to IC-Ach defined as no dilatation or paradoxical vasoconstriction of the coronary artery after the infusion compared to baseline diameter (i.e. abnormal <0% change in coronary artery diameter compared to baseline).

### In subjects with abnormal response to IC-Ach

Seventy-eight subjects (49%) had abnormal response (no dilation or vasoconstriction) to IC-Ach infusion. During CPT, no significant changes were noticed in heart rate, aortic systolic pressure and RPP compared to baseline (P = 0.45, P = 0.053 and P = 0.064 respectively). CBF in response to IC-Ach was poorly correlated to CPT CBF (*r* = 0.17, P = 0.19). At baseline intracoronary blood flow velocity within the LAD was 21.2±8.7 cm/sec and during CPT it was 23.54±9 cm/sec (P = 0.007) in subjects with abnormal response to Ach. The change in coronary artery diameter was -12.25±11.73% in response to Ach while the diameter change was -1.65±10.62% in response to CPT (Table 2). Although statistically significant, the correlation between Ach and CPT diameter change was poor (*r* = 0.353, P = 0.002) (Fig 1B). The change in diameter in response to IC nitroglycerin was 10.97±12.6%.

### Forehead versus forearm cold pressor test

**Fig 2:**
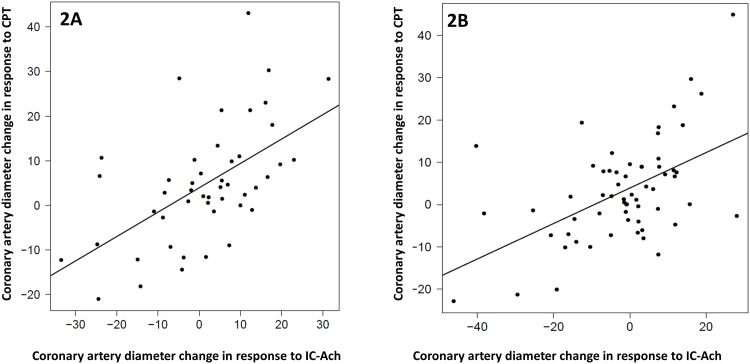
Correlations between coronary artery diameter changes in response to acetylcholine (Ach) and cold pressor testing (CPT)(n = 163). Data are plotted as percent changes from baseline to post Ach or CPT. Subjects are stratified by the CPT type (forehead CPT, 2A, left panel) and (forearm CPT, 2B, right). Note that although correlations among both strata are statistically significant the correlation among those with forehead and forearm CPT is strong (r = 0.56, P<0.001), (r = 0.519, P<0.001) respectively.

**Fig 3:**
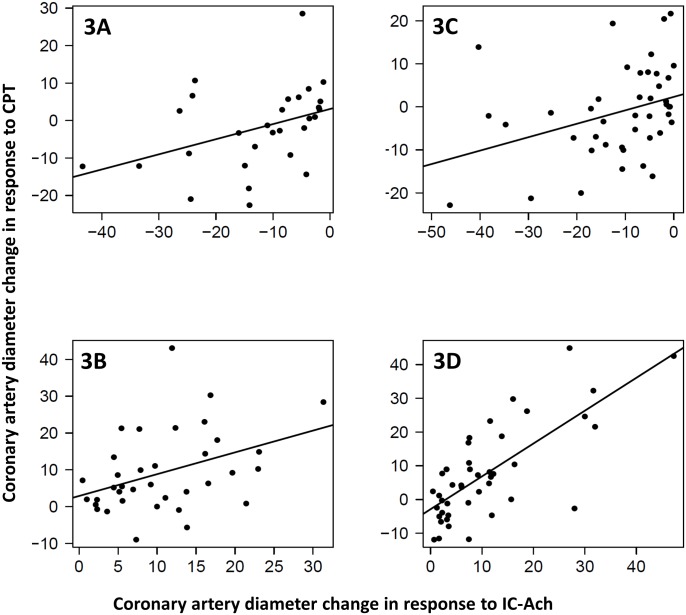
Correlation between acetylcholine and CPT coronary artery diameter changes stratified by endothelial function and CPT type (n = 163). Data are plotted as percent changes from baseline to post Ach or CPT. There was moderate correlation in subjects with *abnormal* endothelial function who underwent forehead CPT, subjects with *normal* endothelial function and forehead CPT and those with *abnormal* endothelial function and forearm CPT (3A: *r* = 0.395, P = 0.33), (3B: r = 0.396, P = 0.017), (3C: r = 0.346, P = 0.23) respectively. There was *strong* correlation in subjects with normal endothelial function who underwent forearm CPT (3D: r = 0.74, P<0.001).

We also evaluated subjects according to the CPT type. Sixty-nine subjects received forehead CPT. There was no significant increase in heart rate, aortic systolic pressure or RPP compared to baseline. However, in subjects who received forearm CPT, we found statistically significant increase in aortic systolic pressure and RPP (4.6±10.7; P<0.001 and 363.3±1338; P = 0.008 respectively). There was no difference in coronary artery diameter change in response to forearm versus forehead CPT (3.45±12.99% versus 4.23±12.29%; P = 0.6). However, there was significant correlation between the coronary artery diameter change in response to either tests and acetylcholine (Fig 2). However, the correlation was stronger in subjects who received forearm CPT and had normal response to Ach (r = 0.74; P<0.001) (Fig 3).

## Discussion

Our results indicate that the normal response to sympathetic stimulation by CPT was mainly coronary dilatation in women with intact endothelium whereas this response varied among the women with endothelial dysfunction defined by Ach infusion. The change in coronary artery diameter was much larger with IC-Ach than with CPT in women with endothelial dysfunction compared to women with normal endothelial function. Coronary vasomotion in response to sympathetic stimulation mirrored the responses to Ach, whereby endothelial dysfunction resulted in a loss of normal coronary dilator function. These results support the suggestion that coronary vasomotion in response to CPT in these women is related to the integrity of endothelial function. Interestingly, the correlation between CBF in response to IC-Ach (which is a measure of microvascular endothelial function) was less correlated to CBF in response to CPT. This might indicate that CPT affects the coronary macrovasculature more than the microvasculature. The microvascular dysfunction is not only a reflection of endothelial function. Passive (structural) and active (smooth muscle tone) both control coronary blood flow in small arteries and arterioles. The flow is affected by the cross-sectional area and the length of the vessels. Furthermore the response to CPT is likely complex—It could be more than just endothelium-related vasodilation, and possibly of use to diagnose coronary vasomotor dysfunction. Specifically, CPT might activate both sympathetic and parasympathetic systems which both have a neuro-hormonal effect on the vessels. In addition, CPT might affect the coronary macro-vasculature more than microvasculature. In addition, the change in coronary artery diameter was much larger with IC-Ach than with CPT in women with endothelial dysfunction compared to women with normal endothelial function, demonstrating that the two provocative tests use differing pathways to test the coronary endothelium.

The diagnosis of endothelial dysfunction is often difficult and requires a specialized center to perform invasive coronary reactivity testing. Invasive evaluation of endothelial function or induction of coronary artery spasm with pharmacologic agents during reactivity testing is rewarding because it provides prognostic information and direction for management.[9] An easily performed and safe provocative test that does not require invasive administration of pharmacologic agents would be of value to evaluate endothelial function in patients with chest pain and no obstructive CAD. We believe that the CPT may offer this potential. The CPT is relatively simple to perform—the response to cold occurs rapidly (approximately 1 minute), is well-tolerated, and is readily reversed by discontinuing the cold stimulus and administering nitroglycerin if needed. The test induces reflex constriction of multiple vascular beds with a consequent increase in peripheral vascular resistance and systemic arterial blood pressure.[17] The CPT provoke angina [18] and has been shown to reduce coronary blood flow in some patients with CAD [12]. While the current results suggest that CPT testing may be of some use for coronary endothelial function testing, particularly among patients with normal endothelial function, they do not support it as an alternative to IC-Ach for diagnosis of coronary endothelial dysfunction.

Previous observations indicated that the normal response to sympathetic stimulation by CPT in patients is coronary vasodilation in patients with normal coronary arteries, but paradoxical constriction may occur in atherosclerotic coronary arteries even at a very early stage of CAD [12]. We found that coronary vasomotion in response to sympathetic stimulation mirrored the effects of the endothelial-dependent vasodilator Ach. Endothelial dysfunction in coronary atherosclerosis likely results in a loss of the normal dilator functions and permits vasoconstrictor responses to sympathetic stimulation. Our results are consistent with previous findings showing that coronary vasomotion in response to CPT in patients is intimately related to the integrity of macrovascular endothelial function [19, 20] although some differences are evident. To understand the differences in our current work and these prior studies, we used a likely less potent CPT which may have led to more variable endothelial dependent vs independent and micro vs macro vessel response. We also used current Doppler flow probes and coronary angiography QCA measures in a blinded core lab, possibly providing more accurate measurements compared to prior decades. We have a larger sample size of 163 compared to prior studies (Montosi, 19), potentially of broader relevance to a more heterogenous population with signs and symptoms of ischemia but no obstructive CAD.

During CPT, the systolic arterial pressure changes we observed were statistically significantly changes compared to baseline in patients with normal endothelial function (P<0.05), while the change was not statistically significant in subjects with endothelial dysfunction. This appears to be mediated by sympathetic activation rather than by parasympathetic withdrawal [21]. In addition, the change in coronary artery diameter was much larger with IC-Ach than with CPT in women with endothelial dysfunction compared to women with normal endothelial function, demonstrating that the two provocative tests use differing pathways to test the coronary endothelium. It is important that arterial pressure be continuously monitored during the procedure because of the possibility of an exaggerated pressor response [22]. Furthermore, others have found that the paradoxical vasoconstrictor response of angiographically “normal”-appearing coronary arteries in patients with evidence of atherosclerosis elsewhere in the coronary system is present as well [23].

The safety of CPT is supported by studies that included patients even with critical multi-vessel atheromatous coronary artery disease. [12, 18] We have, nevertheless, avoided using this test in patients with any important obstructive coronary artery disease. None of our study subjects reports chest pain during the CPT, however it has been reported before and the mechanism was thought to be that the hemodynamic response to CPT (mainly the increase in arterial blood pressure).[17] Hence, chest pain and myocardial ischemia may result from increased myocardial oxygen demand and does not necessarily indicate coronary artery spasm or endothelial dysfunction. Therefore when in doubt the abnormal angiographic response to acetylcholine or demonstration of transient and reversible coronary artery narrowing is still necessary to implicate endothelial dysfunction or spasm. Previously several reports described a link between coronary artery spasm and endothelial dysfunction [24], however the very definition of coronary artery spasm is unsettled. Although it is regarded as a pathologic phenomenon, continuous alteration in vasomotor tone is a normal characteristic of arteries. Alterations in vasomotor tone are to a great extent influenced by the sympathetic nervous system. By stimulating the sympathetic nervous system, the CPT can significantly alter coronary artery diameter, as shown in this study using quantitative arteriography. Unlike our study where the test was performed without complication in patients with suspected endothelial dysfunction, there have been reports of CPT associated with ventricular ectopy and ventricular tachycardia [10].

Previously, CPT was performed by immersing the hand and forearm in ice water (-4°C) for 90 seconds [13]. Although using this technique studies showed there was a significant increase in RPP, we believed that a similar effect could be produced with a less potent stimulus. We found that forearm CPT produces a change in RPP similar to what has been previously reported. Interestingly we did not find this observation with forehead CPT. Perhaps forehead CPT did not produce enough stimulation to increase heart rate and/or systolic blood pressure in addition to the augmented vagal stimulation (diving reflex) [25]. To our knowledge, this is the largest series comparing the effects of Ach to those of CPT invasively. In addition it is the first large study that compared the forehead to forearm CPT. Thus, the results of this study provide evidence that myocardial flow responses to sympathetic activation as assessed invasively may serve as a useful tool for probing predominantly endothelial-dependent coronary vasomotion non-invasively in the future by using alternative imaging techniques.

Some limitations to our findings are worthy of mention. Our subjects were all women with symptoms/signs of ischemic heart disease undergoing clinically-indicated coronary angiography, so it is not possible to extrapolate to other cohorts or determine the specific proportion of all patients with coronary endothelial dysfunction that can be identified by this approach. We did not investigate other angiographic findings that maybe related to endothelial dysfunction, such as perfusion or blush grade. Although the core laboratory was masked to patient information, the cine’s for each subject were labeled indicating when medications or CPT were administered, so the core lab could identify that the cine’s belonged to the same patient. Thus it is possible that core lab reader may have noted changes by one technique and then carried this change, or lack of change, bias over to measurements made with the alternate technique. We found lower correlation between IC-Ach CBF (which is a measurement of microvascular endothelial function) and CPT CBF. Interestingly, this CBF measure did not correlate with adverse outcome in a recent study (470 patients, 68% women) with no obstructive CAD [26].

Finally, we recommend that the CPT be performed before pharmacologic attempts to evaluate endothelial function. Clearly more extensive evaluation in larger cohorts is warranted before the efficacy of CPT as a provocative maneuver for endothelial function is established.

## Conclusions and implications

In women with no obstructive CAD and suspected CMD, coronary diameter changes with IC-Ach and CPT are moderately-well correlated suggesting that CPT testing may be of some use for coronary endothelial function testing, particularly among patients with normal endothelial function, however not an alternative to IC-Ach for diagnosis of coronary endothelial dysfunction. Our study findings indicate that the functional integrity of endothelium appears to be the major determinant of the epicardial coronary artery to the CPT, whereby endothelial dysfunction results in a loss of normal dilator function to sympathetic stimulation in human coronary arteries. CPT is a feasible, easily performed and safe provocative test to evaluate endothelial function in patients with chest pain and no obstructive CAD that does not require administration of pharmacologic agents. In addition, we found that forearm CPT stimulates the sympathetic system more than the forehead CPT test.
